# Antibacterial potential of commercial and wild lactic acid bacteria strains isolated from ovine and caprine raw milk against *Mycoplasma agalactiae*

**DOI:** 10.3389/fvets.2023.1197701

**Published:** 2023-06-22

**Authors:** Marion Toquet, Esther Bataller, Jesús Gomis, Antonio Sánchez, Raquel Toledo-Perona, Christian De la Fe, Juan Carlos Corrales, Ángel Gómez-Martín

**Affiliations:** ^1^Microbiological Agents Associated With Animal Reproduction (ProVaginBIO) Research Group, Departamento Producción y Sanidad Animal, Salud Pública Veterinaria y Ciencia y Tecnología de los Alimentos, Facultad de Veterinaria, Universidad Cardenal Herrera-CEU, CEU Universities, Valencia, Spain; ^2^Ruminant Health Research Group, Departamento de Sanidad Animal, Facultad de Veterinaria, Universidad de Murcia, Murcia, Spain

**Keywords:** *Lactobacillus*, *Enterococcus*, mastitis, contagious agalactia, antimicrobial activity, *Mycoplasma agalactiae*, probiotic, raw milk

## Abstract

**Introduction:**

The complexity of fighting contagious agalactia (CA) has raised the necessity of alternative antimicrobial therapies, such as probiotics. Lactic acid bacteria (LAB) are present in the mammary gland of small ruminants and their antimicrobial effect have been previously described against species like *Mycoplasma bovis* but never against *Mycoplasma agalactiae* (Ma). This *in vitro* study aims to evaluate the antimicrobial activity against Ma of ovine and caprine LAB strains and a human commercial probiotic (L2) of *Lactobacillus* spp.

**Methods:**

A total of 63 possible LAB strains were isolated from nine ovine and caprine farms in Spain, three isolates (33B, 248D, and 120B) from the 63 strains were selected, based on their capacity to grow in a specific medium *in vitro*, for an *in vitro* experiment to assess their antimicrobial activity against Ma in Ultra High Temperature (UHT) processed goat milk (GM). A women commercial vaginal probiotic was also included in the study. The inoculum of L2 was prepared at a concentration of 3.24 × 10^8^  CFU/mL and the average concentration of the inoculum of the wild LAB varied from 7.9 × 10^7^ to 8.4 × 10^8^  CFU/mL.

**Results:**

The commercial probiotic L2 significantly reduced the concentration of Ma to 0.000 log CFU/mL (*p* < 0.001), strain 33B reduced it from 7.185 to 1.279 log CFU/mL (*p* < 0.001), and 120B from 6.825 to 6.466 log CFU/mL (*p* < 0.05). Strain 248D presented a bacteriostatic effect in GM. Moreover, the three wild strains and the commercial probiotic produced a significative reduction of the pH (*p* < 0.001).

**Discussion:**

This is the first *in vivo* report of the antimicrobial potential of LAB strains against Ma and its interaction. Our results support possible future alternative strategies to antibiotic therapy, previously not contemplated, to fight CA in small ruminants. Further studies are necessary to elucidate the action mechanisms through which these LAB are able to inhibit Ma and to assess the safety of using these strains in possible *in vivo* studies.

## 1. Introduction

Contagious agalactia (CA) is an infectious syndrome with an important socioeconomic impact on the small ruminant dairy sector due to negative effects on milk production, premature culling, lessen growth rates, and the high costs of control measures. It is characterized by a triad of clinical manifestations: mastitis, arthritis, and keratoconjunctivitis, but can occasionally affect the reproductive and respiratory tract (1–3). It is a multi-etiological syndrome as four different species from the genus *Mycoplasma* are involved in goats: *Mycoplasma agalactiae* (Ma), *Mycoplasma mycoides* subsp. *capri*, *Mycoplasma capricolum* subsp. *capricolum*, and *Mycoplasma putrefaciens*. Ma is considered as the main etiological agent that affects goats and sheep, as the other three species of mycoplasmas have only been described sporadically as the cause of the disease in the ovine specie (4, 5).

Nowadays, the fight against CA is based on vaccination and antibiotic therapy but the absence of a satisfactory strategy causes difficulties to eradicate CA in endemic regions. In Spain, a national voluntary program based on an accurate diagnosis and the control of the disease has been put in place (6). On one hand, vaccination against CA has its limitation; while commercial vaccines can reduce symptoms and excretion (3), it does not prevent shedding in milk (7) and therefore the carrier state persists (8). Different explanations have been suggested for the lack of an efficient vaccination such as the complex etiology in goats, the high plasticity of the genome of circulating strains or their capacity to evade the immune system (3). In this sense, the development of vaccines that can prevent satisfactorily the infection in flocks or the entrance in areas free of CA does not seem to be a short- and medium-term achievement.

On the other hand, antimicrobial therapy can improve the animals’ health, but it does not eliminate the pathogen (8). It is assumed that antimicrobial agents can reduce the bacterial excretion and clinical symptoms. Nevertheless, the use of antimicrobial agents can generate antimicrobial resistances (AMR), which can compromise the effectiveness of the antimicrobial therapy (3). Indeed, several studies have reported a reduction in the antibiotic susceptibilities of the mycoplasma species associated with CA in different countries (9–16).

In this context surrounding the control and prevention of CA, the necessity to explore alternative therapies, such as the use of probiotics in recent years in people and animals, has emerged. Probiotics are live microorganisms which when administered in adequate amounts confer a health benefit on the host (17). Lactic acid bacteria (LAB) offer various advantages as potential probiotics and can be considered as alternatives to antibiotics (18). They are safe microorganisms able to produce different compounds such as bacteriocins, organic acids as lactic acid, hydrogen peroxide, diacetyl, and carbon dioxide that favor the inhibition of pathogenic microorganisms. Lactic acid bacteria are Gram-positive bacteria, they can be found in the microbiota of various anatomical locations such as the oral cavity, the skin, the gastro-intestinal tract, and the reproductive tract (19–23). Their presence in the raw milk of small ruminants is well known (24), and some strains have been tested *in vitro* for their potential probiotic characteristics (25, 26). As far as we know, LAB isolated in small ruminants have never been challenged against pathogens belonging to *Mycoplasma* spp.

In a previous study, a first dose of a commercial vaginal probiotic for women “L1” was intravaginally inoculated in ewes in order to prevent the vaginosis produced by the use of intravaginal devices. This study reported the capacity of L1 to reduce the vaginal neutrophilia produced by estrus-synchronization sponges without altering the animal health status (27). In addition, a higher dose “L2” of this commercial probiotic has been tested *in vitro* against *Mycoplasma bovis* (Mb) in bovine semen and cervical mucus and showed antimicrobial activity against the pathogen. This antimicrobial activity of *Lactobacillus* spp. was associated to their capacity of acidifying the medium (28, 29). In this sense, the *in vitro* sensitivity of Ma to acid pH has been reported in diluted semen of bucks (30). *Mycoplasma bovis* shares 99% of its genome with Ma (31) and both belong to the hominis group, sharing relevant similarities of intrinsic AMR and therefore control measures (32, 33).

The aim of this microbiological study was to evaluate the *in vitro* antimicrobial potential of lactic acid bacteria, isolated from ovine and caprine raw milk, against *Mycoplasma agalactiae* and compare it with the efficacity of the commercial probiotic L2 dose. To achieve this objective, the viability of *Mycoplasma agalactiae* and lactic acid bacteria as well as the extracellular pH oscillations were evaluated in commercial goat milk and in a *Mycoplasma* spp. specific culture medium.

## 2. Materials and methods

The study design included various steps. The first one was the sampling of 72 animals from nine different farms. The second step involved the isolation of LAB from the raw goat and sheep milk obtained in the first step and the evaluation of their *in vitro* growth capacity in a specific *Mycoplasma* culture medium. The third step consisted in the molecular characterization of the selected strains. The final step was the carrying out of the *in vitro* experiment to assess the antibacterial activity of the different LAB against Ma. All the results from the *in vitro* experiment were statistically analyzed *a posteriori*. In addition, we also analyzed the LAB composition of L2 overtime.

### 2.1. Animals’ description and sampling

Possible LAB strains used in this study (*n* = 63) belong to a collection of the ProVaginBIO investigation group of University CEU—Cardenal Herrera in Valencia, Spain and were isolated from raw milk of ovine (*n* = 48) and caprine animals (*n* = 24), including meat and dairy sheep and goats, from nine different farms (six ovine; three caprine) located in different regions of Spain. The characteristics of the different sampled flocks can be seen in Table 1.

**Table 1:** Characteristics of the different sampled livestock and the selected LAB strains.

Herd	Specie	Breed	Province	Aptitude	G	NIS	NPS	SS	OD	C
A	Caprine	Murciano-Granadina	Castellón	Dairy	No	22	4	33B	0.336	8.4 × 10^8^
B	Ovine	Manchega	Albacete	Meat	Yes	14	8	120B	0.288	3.2 × 10^8^
C	Ovine	Manchega	Albacete	Dairy	Yes	6	0	-	-	-
D	Ovine	Lacaune	Castellón	Dairy	No	4	0	-	-	-
E	Caprine	Negra-Serrana	Valencia	Meat	Yes	2	1	-	-	-
F	Ovine	Guirra	Valencia	Meat	Yes	5	3	248D	0.131	7.9 × 10^7^
G	Ovine	Lacaune	Alicante	Dairy	No	4	0	-	-	-
H	Ovine	Segureña	Jaén	Meat	Yes	0	-	-	-	-
I	Caprine	Murciano-Granadina/Malagueña	Albacete	Dairy	No	6	2	-	-	-

G, grazing; NIS, no. of isolated strains; NPS, no. of potential strains for the experiment; SS, selected strain for the experiment; OD, optical density after 20 h incubation; and C, concentration in CFU/mL after 20 h incubation.

One sheep livestock (herd B) suffered from an outbreak of CA a year before the samples were taken, a reduction in milk production and/or mammary atrophy were observed in 18% of the animals. In this same flock, a strain of Ma with an alarming profile in antibiotic susceptibility tests was isolated. For this reason, antimicrobial therapy was not used. Another flock (herd I) manifested a clinical outbreak during sampling characterized by clinical mastitis, low milk production, and arthritis in kids. In this case, a treatment with tetracyclines was being used in animals showing clinical signs. The use of antibiotics in the other herds was anecdotical.

Prior to the samples collection, a physical examination of the udder was performed through external observation and palpation to rule out the presence of clinical mastitis. A California Mastitis Test (KerbaTEST, KERBL) was also performed prior to collecting milk samples to ensure the animals were not affected by subclinical mastitis. *A posteriori*, all the milk samples were inoculated in a modified specific medium for mycoplasmas growth (34), Columbia agar with 5% sheep blood (BD™) and MacConkey agar (BD™) (27) to rule out the presence of mastitis.

### 2.2. Isolation and selection of lactic acid bacteria

The isolation of LAB was carried out by inoculating the raw ovine and caprine milk samples on Man, Rogosa, and Shape (MRS) agar (Scharlau) (35), and LAB colonies were macroscopically characterized depending on their morphology and frozen at −80°C in cryotubes with 500 μL of liquid MRS and 500 μL of glycerol at 50%.

The 63 isolated strains were tested for their growth in the PH medium. Each strain was activated on MRS agar plates, and one colony was incubated in 4 mL of liquid PH medium at 37°C during 20 h at 150 rpm. Dilutions were performed with phosphate buffer saline solution and four different dilutions were plated on MRS agar. The optical density (OD) was also measured at 600 nm. Strains with OD inferior to 0.100 and with a concentration lower than 10^7^ CFU/mL were discarded to assure an effective scale up yield for a possible industrial production of the selected strain. A total of 18 strains met with the selection criteria and four strains, each from a different type of animal production (dairy goat, meat goat, dairy sheep, and meat sheep), with the highest concentration (CFU/mL) post 20 h incubation and an additional strain isolated from herd I, which had an ongoing CA outbreak at the time of sampling, were selected for molecular characterization previous to the *in vitro* experiment. The final three LAB selected to be tested *in vitro* against Ma can be found in Table 1.

### 2.3. Molecular characterization and bacterial identification of wild LAB strains

The selected strains were characterized, before the *in vitro* experiment. They were processed for genomic DNA extraction and identified based on PCR amplification and sequencing of 16S rRNA gene using bacterial universal primers (27F 5′-AGAGTTTGATCC TGGCTCAG and 1492R 5′-GGTT ACCTTGTTA CGACTT). The PCR was performed following the methodology previously described (21). The PCR products were purified, and sequenced and analyzed for sequence homology by BLAST.^1^ The sequences were corrected and aligned by ClustalW with Molecular Evolutionary Genetics Analysis (MEGA) 7. Bacterial identification was carried out by comparing the problem sequence with the GenBank database through the Blast application.

### 2.4. Design of the *in vitro* experiment

Ten experimental conditions (Table 2) were prepared in Eppendorf-type tubes of 1.5 mL capacity following an adaption of a previous protocol (28, 29). An eleventh (C11) and twelfth (C12) microtubes were included as negative controls. Each wild LAB strain (LX) was tested in three independent replicates of the experimental conditions. The conditions were incubated for 15 h.

**Table 2:** Composition of the experimental conditions.

Condition	Composition
1	GM (1,460 μL) + Ma (40 μL)
2	GM (1,000 μL) + L2 (500 μL)
3	GM (960 μL) + Ma (40 μL) + L2 (500 μL)
4	GM (1,000 μL) + LX (500 μL)
5	GM (960 μL) + Ma (40 μL) + LX (500 μL)
6	PH (1,460 μL) + Ma (40 μL)
7	PH (1,000 μL) + L2 (500 μL)
8	PH (960 μL) + Ma (40 μL) + L2 (500μL)
9	PH (1,000 μL) + LX (500 μL)
10	PH (960 μL) + Ma (40 μL) + LX (500 μL)
11	GM (1,500 μL)
12	PH (1,500 μL)

GM, semi-skimmed UHT goat milk; Ma, *Mycoplasma agalactiae* strain PG2; L2, commercial probiotic inoculum; LX, ovine/caprine lactic acid bacteria inoculum for each selected strain (33B, 248D, and 120B); and PH, specific medium for *Mycoplasma* spp. growth.

#### 2.4.1. Preparation of *Mycoplasma agalactiae* inoculum

The Ma inoculum was prepared using the reference strain (PG2, NCTC10123) in PH medium with ampicillin and following the protocol previously described (28, 29). The culture was incubated at 37°C during 48 h, then a subculture was realized and incubated 48 h at 37°C again to obtain our inoculum with an approximate concentration of 1 × 10^7-8^CFU/mL, based on previous inoculations and the infective dose of Ma (30), and calculated as previously described (36).

#### 2.4.2. Preparation of wild ovine/caprine lactic bacteria inoculum

The ovine/caprine LAB inoculum (LX) consisted of the culture of a single colony of each of the selected LAB strains, previously isolated from raw milk, in 4 mL of PH medium without any added antibiotics at 37°C for 20 h. The tubes were then centrifugated at 4,000 rpm for 15 min. The supernatant was discarded, and the precipitate was reconstituted in microtubes of 1.5 mL with 500 μL of PH medium without antibiotics. The average concentration of the inoculum LX varied from 7.9 × 10^7^ to 8.4 × 10^8^ CFU/mL.

#### 2.4.3. Preparation of L2 inoculum

The inoculum of the commercial probiotic (L2) was prepared at a concentration of 3.24 × 10^8^ CFU/mL as previously described (28, 29). A capsule of a commercial probiotic based on a mix of *Lactobacillus crispatus*, *Lactobacillus gasseri*, and *Lactobacillus brevis* (NS Femibiotic®, Cinfa) was reconstituted in PH medium.

#### 2.4.4. Determination of *Mycoplasma agalactiae* and lactic acid bacteria viability

Concentrations (CFU/mL) of Ma and LAB were determined after 15 min (T0) and 15 h (T15). The Ma viability was determined with a protocol of serial dilutions previously described (36) using PH broth supplemented with ampicillin for serial dilutions and PH agar supplemented with ampicillin for bacterial counts (34). The LAB viability was determined on MRS agar plates, with dilutions also performed in PH broth. Every dilution was plated in duplicate.

#### 2.4.5. pH measurement

The pH of every condition was measured with a calibrated pH-meter (SensION™ + pH3, Hach, LPV2000.98.0002) at T0 and T15. The electrode was disinfected with detergent, alcohol and sterile distilled water between the measurement of each condition to avoid contamination.

### 2.5. Statistical analysis of pH, lactic acid bacteria, and *Mycoplasma agalactiae* viability

Counts of Ma and LAB were transformed as log (1 + C), where C was the count obtained (CFU/mL) for each analytical condition and organism. Statistical analysis was performed using a general linear procedure implemented in the program Statistical Analysis System Institute (SAS), following the model: Y_ijk_ = μ + S_i_ + C_j_ + T_k_ + CT_jk_ + e_ijk_, where Y_ijk_ = pH and log CFU/mL of Ma and log CFU/mL of LAB in each strain studied (33B, 120B, and 248D); μ = mean; S_i_ = sample effect; C_j_ = effect of analytical conditions; T_k_ = effect of time; CT_jk_ = effect of the interaction between the analytical condition and time; and e_ijk_ = residual effect.

### 2.6. Microbial composition of L2 at T0 and T15

A marker-based approach using the 16S ribosomal RNA subunit gene (16SrRNA) was used to confirm the *Lactobacillus* spp. present in L2 and to study their fluctuation in condition 2 (C2) at T0 and T15, condition 3 (C3) at T0 and T15, condition 7 (C7) at T0 and T15, and condition 8 (C8) at T0 and T15.

The composition and structure of the sampled microbial communities was assessed through the amplification and sequencing the V3-V4 variable regions of the 16S rRNA gene. The Illumina Miseq sequencing 300 × 2 approach was used. Amplification was performed after 25 PCR cycles. A negative control of the DNA extraction was included as well as a positive Mock Community control to ensure quality control. Raw demultiplexed forward and reverse reads were processed as shown in the following Table 3 using QIIME2 (40).

**Table 3:** Processing of raw demultiplexed forward and reverse reads.

Step	Methods used
1. Primer trimming	Dada2 (37)
2. Quality filtering	Dada2
3. Denoising	Dada2
4. Pair-end merging	Dada2
5. Phylotype calling	Dada2
6. Phylogeny assessment	Mafft and Fasttree (38, 39) PRINTDATE \* MERGEFORMAT

Taxonomic assignment of phylotypes was performed using a Bayesian Classifier trained with Silva database version 138 (99% OTUs full-length sequences) (41).

## 3. Results

### 3.1. Identification of wild lactic acid bacteria strains

Based on the sequences obtained, strain 33B was identified as *Enterococcus mundtii* (OQ538168), strain 120B as *Enterococcus hirae* (OQ538169) and strain 248D as *Enterococcus hirae* (OQ538170), and the GenBank submission number being SUB12912028. The other two strains that were selected for molecular characterization were both identified as *Staphylococcus aureus* subsp. *aureus* and were therefore not included in the *in vitro* experiment.

### 3.2. *In vitro* experiment negative controls

The conditions C11 (GM) and C12 (PH) always came back negative on sheep blood agar plates, MRS agar plates and PH agar plates at T0 and T15. The average pH of C11 at T0 and T15 ranged from 6.61 to 6.69, respectively, and the average pH of C12 at T0 and T15 ranged from 7.51 and 7.68.

### 3.3. Effects on *Mycoplasma agalactiae* and lactic acid bacteria viability and pH

In the *in vitro* proposed model, and for each LAB strain studied, the condition itself, the time and the interaction between condition and time had a significant effect (*p* < 0.001) on the pH and the log CFU/mL of Ma. The factor condition contributed significantly to the observed log CFU/mL of LAB variation in all the LAB strain studies, while the factors time and the interaction between condition and time contributed significantly for the LAB strain 33B and 120B, and for 248D, respectively.

#### 3.3.1. Strain 33B

Table 4 details the evolution of the pH and the viability of Ma and LAB over time for the experiment with strain 33B. In favorable conditions, condition 1 (C1) and condition 6 (C6), Ma concentration did significantly increase, and the pH showed stable values between T0 and T15. The strain 33B produced a statistically significant decrease of the concentration of Ma in GM [condition 5 (C5)] and PH medium [condition 10 (C10)]. The pH decreased significantly (*p* < 0.001) in GM in presence of the 33B strain [condition 4 (C4) and C5] between T0 and T15, but it did not in PH medium [condition 9 (C9) and C10] although it was statistically significantly lower in C9-C10 compared to C12. No differences were observed between T0 and T15 for the concentration of LAB.

**Table 4:** Least squares means of pH and log CFU/mL of Ma and LAB by time for the strain 33B.

Condition	Composition	Time	Ma (LOG CFU/mL)^1^	LAB (LOG CFU/mL)^2^	pH^3^
1	GM + Ma	0	7.248^ab^	-	6.59^gh^
1	GM + Ma	15	7.793^ab^	-	6.50^h^
2	GM + L2	0	-	8.760^a^	6.22^j^
2	GM+ L2	15	-	8.743^a^	4.09^l^
3	GM + Ma + L2	0	7.083^b^	8.806^a^	6.35^i^
3	GM + Ma + L2	15	0.000^e^	8.714^ab^	4.20^l^
4	GM + 33B	0	-	8.465^abcd^	6.55^gh^
4	GM + 33B	15	-	8.217^cde^	5.29^k^
5	GM+ Ma + 33B	0	7.185^ab^	7.675^g^	6.55^gh^
5	GM + Ma + 33B	15	1.279^d^	7.789^g^	5.34^k^
6	PH + Ma	0	7.069^b^	-	7.47^b^
6	PH + Ma	15	8.015^a^	-	7.29^c^
7	PH + L2	0	-	8.595^abc^	6.79^f^
7	PH + L2	15	-	8.257^bcde^	6.95^de^
8	PH + Ma + L2	0	7.138^ab^	8.803^a^	6.81^f^
8	PH + Ma + L2	15	7.126^ab^	8.424^abcd^	6.89^def^
9	PH + 33B	0	-	8.257^bcde^	6.86^ef^
9	PH + 33B	15	-	7.873^efg^	7.00^d^
10	PH + Ma + 33B	0	7.111^ab^	8.059^def^	6.87^ef^
10	PH + Ma + 33B	15	2.209^c^	7.806^fg^	6.84^ef^
11	GM	0	-	-	6.62^gh^
11	GM	15	-	-	6.65^g^
12	PH	0	-	-	7.48^b^
12	PH	15	-	-	7.64^a^

GM, semi-skimmed UHT goat milk; Ma, *Mycoplasma agalactiae* strain PG2; L2, commercial probiotic inoculum; 33B, lactic acid bacteria strain 33B inoculum; PH, specific medium for *Mycoplasma* spp. growth; LAB, lactic acid bacteria; ^1^SEM: 0.33; ^2^SEM: 0.16; ^3^SEM: 0.04. ^a–l^Means in the same column with different superscripts differ significantly (*p* < 0.05).

#### 3.3.2. Strain 248D

Table 5 details the evolution of the pH and the viability of Ma and LAB over time for the experiment with strain 248D. In favorable conditions (C1 and C6), Ma concentration significantly increased, and the pH showed stable values between T0 and T15. The concentration of strain LAB 248D significantly increased with the presence of Ma in GM (C5). Although it did not reduce the concentration of Ma in GM (C5), it was able to prevent the proliferation of Ma between T0 and T15 as the concentration of Ma did not increase in C5 and it was significantly lower than C1 at T15. The strain 248D was also able to significantly decrease the pH over time in GM (C4-5) although without the presence of Ma (C4) the pH was significantly lower at T15 compared to C5. On the other hand, in the PH medium the concentration of LAB 248D significantly decreased at T15 (C9-10) and Ma increased significantly at T15 with the presence of the strain 248D (C10). The pH of the PH medium was stable over time although the conditions with LAB (C7-10) had a pH significantly lower compared to C6 and C12.

**Table 5:** Least squares means of pH and log CFU/mL of Ma and LAB by time for the strain 248D.

Condition	Composition	Time	Ma (LOG CFU/mL)^1^	LAB (LOG CFU/mL)^2^	pH^3^
1	GM + Ma	0	7.020^d^	-	6.59^d^
1	GM + Ma	15	8.030^a^	-	6.57^d^
2	GM+ L2	0	-	8.878^cd^	6.42^d^
2	GM + L2	15	-	9.276^a^	4.11^g^
3	GM + Ma + L2	0	6.928^de^	8.681^de^	6.45^d^
3	GM + Ma + L2	15	0.000^f^	9.227^ab^	4.21^g^
4	GM + 248D	0	-	8.635^de^	6.51^d^
4	GM + 248D	15	-	8.800^cde^	4.82^f^
5	GM + Ma + 248D	0	6.883^de^	8.584^e^	6.54^d^
5	GM + Ma + 248D	15	6.711^e^	8.999^bc^	5.14^e^
6	PH + Ma	0	6.822^de^	-	7.47^ab^
6	PH + Ma	15	7.949^ab^	-	7.33^b^
7	PH + L2	0	-	8.663^def^	6.86^c^
7	PH + L2	15	-	8.642^defg^	6.82^cd^
8	PH + Ma + L2	0	6.834^de^	8.664^def^	6.84^c^
8	PH + Ma + L2	15	7.382^c^	8.693^de^	6.79^cd^
9	PH + 248D	0	-	8.778^cde^	7.00^c^
9	PH + 248D	15	-	8.418^fg^	6.94^c^
10	PH + Ma + 248D	0	7.070^d^	8.761^cde^	6.99^c^
10	PH + Ma + 248D	15	7.719^b^	8.388^g^	6.77^cd^
11	GM	0	-	-	6.58^d^
11	GM	15	-	-	6.58^d^
12	PH	0	-	-	7.52^ab^
12	PH	15	-	-	7.63^a^

GM, semi-skimmed UHT goat milk; Ma, *Mycoplasma agalactiae* strain PG2; L2, commercial probiotic inoculum; 248D, lactic acid bacteria strain 248D inoculum; PH, specific medium for *Mycoplasma* spp. growth; LAB, lactic acid bacteria; ^1^SEM: 0.10; ^2^SEM: 0.09; and ^3^SEM: 0.08. ^a–g^Means in the same column with different superscripts differ significantly (*p* < 0.05).

#### 3.3.3. Strain 120B

Table 6 details the evolution of the pH and the viability of Ma and LAB over time for the experiment with strain 120B. In favorable conditions (C1 and C6), Ma concentration significantly increased, and the pH showed stable values between T0 and T15. In presence of strain 120B, a significant decrease in the concentration of Ma can be observed between T0 and T15 in GM, associated with a significantly reduction of the pH (C5). This was not the case in PH medium (C10), where the concentration of Ma significantly increased at T15 associated with a stability in LAB concentration and pH.

**Table 6:** Least squares means of pH and log CFU/mL of Ma and LAB by time for the strain 120B.

Condition	Composition	Time	Ma (LOG CFU/mL)^1^	LAB (LOG CFU/mL)^2^	pH^3^
C1	GM + Ma	0	6.798^d^	-	6.65^d^
C1	GM + Ma	15	7.726^a^	-	6.68^cd^
C2	GM + L2	0	-	8.713^abcde^	6.36^de^
C2	GM + L2	15	-	9.018^ab^	4.19^g^
C3	GM + Ma + L2	0	6.774^d^	9.119^a^	6.35^e^
C3	GM + Ma + L2	15	0.000^f^	8.936^abc^	4.27^g^
C4	GM + 120B	0	-	8.576^bcde^	6.64^d^
C4	GM + 120B	15	-	8.728^abcd^	5.50^f^
C5	GM + Ma + 120B	0	6.825^d^	8.376^def^	6.62^d^
C5	GM + Ma + 120B	15	6.466^e^	8.490^cde^	5.43^f^
C6	PH + Ma	0	6.689^de^	-	7.57^a^
C6	PH + Ma	15	7.424^b^	-	7.71^a^
C7	PH + L2	0	-	8.614^bcde^	6.92^bc^
C7	PH + L2	15	-	8.667^bcde^	6.90^bc^
C8	PH + Ma + L2	0	6.928^cd^	8.789^abcd^	6.90^bc^
C8	PH + Ma + L2	15	6.858^d^	8.523^cde^	6.93^bc^
C9	PH + 120B	0	-	8.570^bcde^	7.07^b^
C9	PH + 120B	15	-	7.951^f^	7.08^b^
C10	PH + Ma + 120B	0	6.838^d^	8.669^abcde^	7.04^b^
C10	PH + Ma + 120B	15	7.154^c^	8.276^ef^	7.02^b^
C11	GM	0	-	-	6.62^d^
C11	GM	15	-	-	6.83^bcd^
C12	PH	0	-	-	7.53^a^
C12	PH	15	-	-	7.78^a^

GM, semi-skimmed UHT goat milk; Ma, *Mycoplasma agalactiae* strain PG2; L2, commercial probiotic inoculum; 120B, lactic acid bacteria strain 248D inoculum; PH, specific medium for *Mycoplasma* spp. growth; LAB, lactic acid bacteria; ^1^SEM: 0.09; ^2^SEM: 0.16; ^3^SEM: 0.10.

^a–g^Means in the same column with different superscripts differ significantly (*p* < 0.05).

#### 3.3.4. Commercial probiotic (L2)

The commercial inoculum L2 was able to completely inhibit Ma in GM as no colonies were observed at T15 in any of three replicas of the three wild LAB strains (C3 in Tables 4–6). The concentration of LAB was similar at T0 and T15 in every experiment except for strain 248D (C2-3, Table 5) where a significant increase of concentration of LAB was observed at T15 in GM. The pH in GM was significantly reduced in all the experiments (C2-C3, Tables 4–6) when L2 was added. No pH reduction was observed between T0 and T15 in PH medium conditions (C7-8 in Tables 4–6), where L2 is present. Nevertheless, there was a significative difference between the pH of medium PH without any LAB (C6 and C12) and C7 and C8 at T0 (Tables 4–6).

### 3.4. Lactic acid bacteria composition of L2 per condition and time

**Figure 1:**
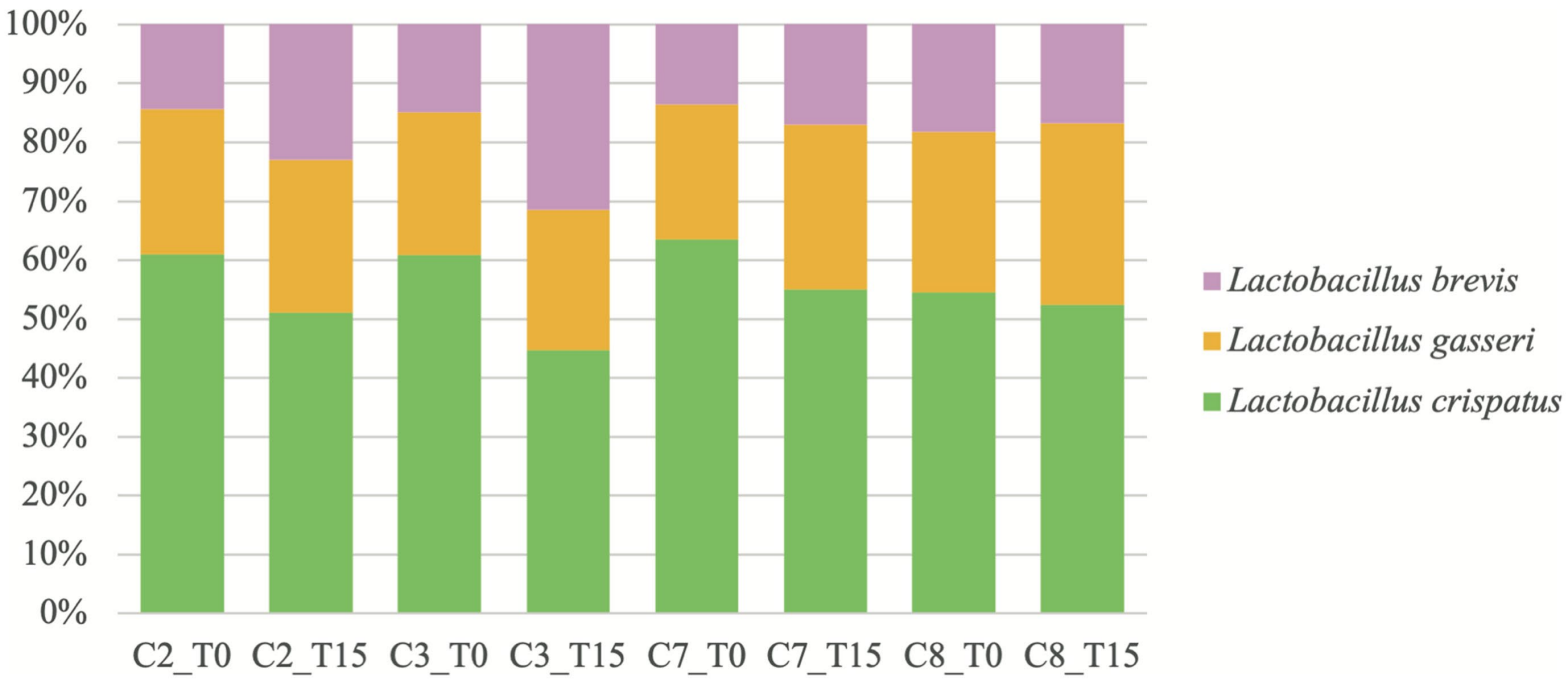
Relative abundances, reported as percentages, of *Lactobacillus* spp. over time in conditions where L2 is present. C2: condition 2 with goat milk and L2; C3: condition 3 with goat milk, *Mycoplasma agalactiae* PG2 and L2; C7: condition 7 with PH medium and L2; C8: condition 8 with PH medium, *Mycoplasma agalactiae* PG2 and L2; T0: after 15 min incubation; and T15: after 15 h incubation.

Metagenomic analysis (Figure 1) revealed that the three LAB species in conditions containing L2 (C2-C3 and C7-C8) were *Lactobacillus crispatus, Lactobacillus gasseri*, and *Lactobacillus brevis*, as described by the manufacturer, at both T0 and T15. *Lactobacillus crispatus* was always the most abundant LAB specie in all the conditions mentioned at T0 and T15, although its relative abundance (RA) decreased at T15 in every condition, with a RA > 50% except at T15 in GM with the presence of Ma (C3). *Lactobacillus gasseri* was the second most abundant of all three species at both times except at T15 in C3. The RA of *L. gasseri* at T15 stayed similar in GM (C2-C3) but increased in PH medium (C7-C8). Finally, *L. brevis* was always the least abundant specie, except in C3 at T15 where it was more abundant than *L. gasseri*. Its RA increased at T15 in every condition apart from C8.

## 4. Discussion

The present *in vitro* study reports the antimicrobial effect against Ma of a selection of wild LAB isolates from the milk of healthy sheep and goats. These strains were isolated in herds located in the mainland of Spain (Table 1), an area where etiological agents associated with CA have been frequently isolated in ovine and caprine species (4, 42). Contagious agalactia control and prevention represent a challenge due to several factors: presence of asymptomatic carriers, uncontrolled movement of animals, variability in etiology and antigenicity, the limitations of commercially available vaccines and the increasing AMR of mycoplasmas associated with CA (1–3, 10). Our results suggest that the commercial probiotic used in this study, based on a combination of *Lactobacillus* spp., or wild LAB of ovine and caprine origin could have the potential of being used as antimicrobials for the control or prevention of mastitis caused by Ma.

Our work evinces that an important number of bacterial isolations is necessary in various flocks to obtain LAB strains capable of growing in a culture medium that allow their *in vitro* testing and demonstrate a possible commercial use (Table 1). All the three wild strains of LAB involved in the *in vitro* experiments were able to inhibit the growth of Ma in GM. Strain 248D had a bacteriostatic effect as it did not significantly decrease the number of Ma at T15, but it did prevent its ease to replicate and increase its concentration in GM at 37°C (Table 5, C5 and C1). Strains 33B and 120B were able to significantly reduce the concentration of Ma at T15 in GM (Tables 4, 6; C5) although the inhibition by 33B was significantly greater than the inhibition produced by 120B (*p* < 0.001).

Lowering the pH is an important feature of LAB as it can inhibit the growth of pathogenic bacteria (43). The acidification of the medium has been suggested to inhibit Ma and *M. mycoides* subsp. *capri*. in diluted semen of bucks as these species are sensitive to pH changes (30). Therefore, one of the causes of the inhibition produced by these LAB may be the drop in the pH of the GM they produced which does not occur when the GM only carries Ma and so the pathogen increases its concentration (C1). All the wild ovine and caprine strains tested in the *in vitro* experiments were able to acidify the GM (Tables 4–6; C4) as there was a significant difference between the GM pH of T0 and T15.

Nevertheless, the strain 33B, identified as *E. mundtii*, was able to inhibit Ma in PH medium (Table 4, C10) with a pH close to neutral and could therefore show better antimicrobial capacity than L2 (Tables 5, 6, C8) in environments where the pH is neutral, and the acidification of the medium is not possible. This suggests that pH acidification may not be the only antimicrobial effect of LAB against Ma and that other antimicrobial mechanisms should be sought.

Probiotics bacteria have several mechanisms of action to inhibit pathogenic bacteria *in vivo*: competing for nutrients, preventing the adhesion of the pathogens, producing inhibitory substances, modulating the host immune response, and reducing the bioavailability of toxins (18). It is unlikely that LAB and Ma compete for the same nutrients given that LAB use glucose to produce lactic acid (44) and Ma cannot ferment glucose unlike other species such as *M. mycoides* subsp. *capri*, *M. capricolum* subsp. *capricolum* and *M. putrefasciens* (30). Therefore, we propose the hypothesis that these bacteria could have a greater inhibitory effect against sugar-fermenting mycoplasma species. In the case of Ma, the production of inhibitory substances *in vitro* could be one of the antimicrobial mechanisms used by LAB, in addition to the harmful effect produced by acidification of the extracellular pH, given that the inhibition in PH medium (Table 4, C10) occurred without a medium acidification for the strain 33B.

One of the inhibitory substances produced by LAB are bacteriocins, and raw milk can be considered as a source of LAB strains with bacteriogenic potential (45, 46). The *E. mundtii* strain CRL 1656 isolated from cow’s milk has been reported as bacteriocin-producing strain and showed a bacteriocigenic activity against the pathogen *Listeria monocytogenes* Scott A and *L. innocua* 7. This strain also able to produce a good amount of hydrogen peroxide, another inhibitory substance produced by LAB. Its use as a probiotic in cows has been recommended (46). Another strain, *E. mundtii* EM ML2/2, isolated from raw goat milk, produced a bacteriocin substance and showed an optimal activity at pH 6.3 (47).

The two other LAB strains, both isolated from meat sheep, with a bacteriostatic (248D) and bactericidal (120B) potential were identified as *Enterococcus hirae*. These results evince that different antimicrobial effects against Ma can be observed for different strains of same LAB specie. Other strains of *E. hirae* ST57ACC and DF105Mi have shown antimicrobial activity against *L. monocytogenes* by producing bacteriocins capable of resisting food processing (25, 45). A strain isolated from GM was also able to modulate the gut microbiota in dogs and did not present any virulence gene (43).

Regarding the evaluation of the commercial probiotic, the addition of L2 in GM (C3) showed a significantly higher bactericidal activity (*p* < 0.001) against Ma than that observed with strain 33B and 120B in GM (Tables 4, 6; C2-C5). This could also be related to the significant pH decrease observed throughout all experiments. Indeed, L2 significantly reduced the pH of the GM below five, when with Ma (Tables 4–6, C3), while strains 33B, 120B, or even 248D lowered the pH to values between 5.14 and 5.43 (Tables 4–6, C5). In previous studies, a similar inoculum was evaluated *in vitro* against Mb in bovine diluted semen and cervical mucus of cattle, and a significant reduction in the pH was also observed (28, 29). Consistent with these studies, our results showed that L2 can also grow and acidify the extracellular medium in GM even when contaminated by Ma and could be a tool used as an antimicrobial strategy as it has been suggested (30). However, as mentioned previously, other possible influences such as competition for nutrients or the possible presence of bioactive peptides should not be ruled out as an antimicrobial mechanism of LAB (18).

These data regarding L2 could show a possible increase in the antimicrobial potential against Ma when several species of *Lactobacillus* spp. are used together as probiotics in GM. In this sense, the combination of various LAB strains is usually employed in commercial probiotics due to their synergy that increases their biological activity (48). The exact composition of this inoculum or one of similar composition had not been evaluated in previous studies (28, 29). In the present study, metagenomic analysis of the conditions with L2 evidenced for the first time, the real composition of this inoculum developed from a commercial probiotic for human use. The results showed that indeed, three species of *Lactobacillus* spp. are inoculated with our protocol (Figure 1). Our metagenomic study of the dynamics of the three species of *Lactobacillus* spp. of L2 showed that *L. brevis* increased its concentration to the detriment of *L. crispatus* in GM contaminated with Ma or not while *L. gasseri* had a steady RA over time. This provides a first approximation of the dynamics of these lactobacilli species in two different media and the possible role of *L. brevis* in the inhibition of Ma in GM. This specie has been isolated in raw milk of goats (49) and seems to have an antimicrobial effect against several pathogens such as *Bacillus cereus* (50), *Escherichia coli, S. aureus*, *Klebsiella pneumoniae*, and *Pseudomonas aeruginosa* (51). Nevertheless, it was reported that *L. brevis* was unable to acidify milk during a 20.5 h fermentation at 37°C (50) and could therefore not be responsible for the significantly lower pH observed at T15 in GM (C2-C3) and use different mechanisms to inhibit pathogens. *Lactobacillus gasseri* has also been isolated in caprine raw milk (52, 53) and seems to be a main component of the human vaginal flora, as well as *L. crispastus* (54), although the latest has never been isolated in milk to our knowledge.

Generally, this work suggests the antimicrobial potential of LAB against Ma under *in vitro* conditions, an important pathogen of the mammary gland of small ruminants. The necessity to explore possible applications of LAB, present in the microbiota of the mammary gland, as a control and prevention strategy against small ruminants’ mastitis was previously suggested (24). Different studies have demonstrated the positive effect of LAB and its metabolites on the welfare of farm animals. It has been shown that the use of probiotics based on LAB reduces the occurrence of pathogens in large-scale farms (55). The results of the present *in vitro* study would suggest the need to inoculate, *in vivo* in caprine and ovine models, the strains identified in this study with an antimicrobial potential against Ma. In this sense, a preliminary study developed an intravaginal inoculation method in ewes, with doses inferior to L2 of the commercial probiotic used in this study, which showed the first signs of anti-inflammatory effects and had no prejudicial effects on the animals’ health (27).

On the other hand, from an epidemiologic point of view, our results show that LAB with a negative effect against Ma can be naturally present in the mammary gland of ewes (248D, 120B) and goats (33B) from endemic regions of CA (Table 1). In all the three herds where the strains with antimicrobial potential were isolated, the use of antibiotics was anecdotic. The herds where 33B and 248D were isolated did not have any CA outbreaks, at least in the last decade, although they did manifest symptoms compatible with CA in the past. On the contrary, the ovine flock where 120B was isolated, had a clinical history of CA a year before this study took place. It is known that after a clinical outbreak of CA, the affected herds usually become chronically infected. This is normally attributed to an equilibrium created between the host and the pathogen, depending on the immune status of the herd. Moreover, it is accepted that the infection is not usually eliminated after the use of antibiotics and vaccines (2, 56, 57). Our results show the existence of LAB with antimicrobial potential against Ma in a CA chronically infected herd (strain 120B, Herd B, Table 1). Curiously, approximately one year after of this isolation, a new episode of decreased milk production was observed in this herd in animals where Ma was isolated again but no LAB was isolated. Therefore, the isolation of LAB never coincided with that of Ma and vice versa. In the herd that had a clinical outbreak of CA at the time of this study (Table 1, herd I), LAB were not isolated either. We suggest the hypothesis that this type of bacterial population (LAB) could contribute to the maintenance of the apparent asymptomatic status of a high number of animals in infected flocks. Furthermore, we need to consider that pathogenic species of *Mycoplasma* in ruminants such as the ones associated to CA (3, 58, 59), in asymptomatic animals, are usually found in anatomic locations such as articular liquid, lymph nodes, brain or external auditive canal, perpetuating the infection in the herds. We propose that with this strategy the pathogens not only try to avoid the immune system and the antimicrobial therapy (1, 2) but also the cohabitation with bacterial groups with antimicrobial potential such as the LAB. Indeed, these LAB populations can be found in the microbiota of the epithelium of the respiratory, mammary and reproductive tracts (23, 24, 60), which are anatomical locations that are colonized by mycoplasma associated with CA and linked to excretion route (34). In this sense, in a previous study involving *Salmonella* sp., the isolation of LAB was less important in dogs that were positive to this pathogen (21). Based on this hypothesis, the use of antibiotics could harm the natural barrier, that LAB with antimicrobial capacity represent, in locations such as the mammary gland of small ruminants. The results reported here could be the first indication of an undervalued interaction of LAB with other microbial agents, such as Ma, and suggests the need to carry out new studies on the bacterial ecology in CA infected animals.

In conclusion, this study marks the first description of the antimicrobial potential of LAB against Ma, hence a possible new alternative to the antibiotics used for the control of CA. To the authors’ knowledge, the assessment of the antimicrobial potential of wild LAB against mycoplasmas of the hominis group has not previously been reported. In this sense, the inoculum L2, elaborated from a human commercial probiotic based on *Lactobacillus* spp., evinces itself as a strategy capable of achieving the complete inhibition of Ma *in vitro* in GM. The presence of *E. hirae* and *E. mundtii* is also confirmed in ovine and caprine milk with an *in vitro* bacteriostatic or bactericidal capacity against Ma in milk. The interaction between LAB and Ma reported here suggests a possible role of LAB in the dynamics of mycoplasmosis that should be studied. Our results suggest the necessity to design further *in vitro* studies to characterize other aspects of these LAB strains, such as other functional properties, bio-preservation and safety, as well as try to understand the inhibitory mechanisms, in order to corroborate their probiotic potential. In addition*, in vivo* studies would be needed to confirm its antimicrobial potential against mycoplasmas associated with CA and its innocuity on animals’ health.

## Data availability statement

The datasets presented in this study can be found in online repositories. The names of the repository/repositories and accession number(s) can be found at: https://www.ncbi.nlm.nih.gov/; OQ538168, OQ538169, and OQ538170.

## Author contributions

MT, EB, and ÁG-M designed the study and wrote the manuscript with input from all authors. MT, JG, RT-P, and ÁG-M collected the samples. MT and RT-P processed and analyzed the samples. MT, EB, JG, RT-P, and ÁG-M performed the laboratory experiments. MT recollected and prepared the data. AS analyzed the data. MT, EB, AS, JC, CF, and ÁG-M interpreted the data. All authors contributed to the article and approved the submitted version.

## Funding

This work was supported by Generalitat Valenciana (Spain; GVA/2020/026) and the Spanish Ministry of Science and Innovation (PID2020-119462RA-I00/AEI/10.13039/501100011033). MT is supported by a pre-doctoral contract of the CEU-UCH, RT-P by a pre-doctoral contract of the Generalitat Valenciana (CIACIF/2021/245), and ÁG-M by a “Ramón y Cajal” contract of the Spanish Ministry of Science and Innovation (RYC2021-032245-I).

## Conflict of interest

The authors declare that the research was conducted in the absence of any commercial or financial relationships that could be construed as a potential conflict of interest.

## Publisher’s note

All claims expressed in this article are solely those of the authors and do not necessarily represent those of their affiliated organizations, or those of the publisher, the editors and the reviewers. Any product that may be evaluated in this article, or claim that may be made by its manufacturer, is not guaranteed or endorsed by the publisher.

## Footnotes

^1^ http://www.ncbi.nlm.nih.gov/
